# Reduced circulating BMP9 and pBMP10 in hospitalized COVID‐19 patients

**DOI:** 10.1002/pul2.12192

**Published:** 2023-01-27

**Authors:** Benjamin J. Dunmore, Paul D. Upton, Kate Auckland, Romit J. Samanta, CITIID‐NIHR BioResource COVID‐19 Collaboration, The EpiCov Database, Paul A. Lyons, Kenneth G. C. Smith, Stefan Gräf, Charlotte Summers, Nicholas W. Morrell

**Affiliations:** ^1^ Heart and Lung Research Institute University of Cambridge Cambridge UK; ^2^ Department of Medicine University of Cambridge School of Clinical Medicine Cambridge UK; ^3^ Cambridge University Hospitals and Royal Papworth Hospital NHS Foundation Trust Cambridge UK; ^4^ NIHR BioResource for Translational Research Cambridge University Hospitals NHS Foundation Trust Cambridge UK; ^5^ NIHR Biomedical Research Center Cambridge University Hospitals Foundation Trust and University of Cambridge Cambridge UK; ^6^ Cambridge Institute of Therapeutic Immunology and Infectious Disease, Jeffrey Cheah Biomedical Centre University of Cambridge Cambridge UK

**Keywords:** BMPs, endothelial cell dysfunction, viral infections and pathogenesis

## Abstract

Similar to other causes of acute respiratory distress syndrome, coronavirus disease 2019 (COVID‐19) is characterized by the aberrant expression of vascular injury biomarkers. We present the first report that circulating plasma bone morphogenetic proteins (BMPs), BMP9 and pBMP10, involved in vascular protection, are reduced in hospitalized patients with COVID‐19.

## INTRODUCTION

The rapid spread of a severe acute respiratory syndrome coronavirus (SARS‐CoV‐2), first identified in 2019, led to a major worldwide public health crisis. For many individuals, coronavirus disease 2019 (COVID‐19) led to no or mild symptoms. However, for some patients, severe COVID‐19 resulted in respiratory failure, admission to intensive care, the requirement for mechanical ventilation, and death. According to the World Health Organization (WHO), infection with SARS‐CoV‐2 was observed in over 600 million individuals worldwide and accounted for more than 6 million deaths by October 2022.

Originally, SARS‐CoV‐2 was reported to directly infect pulmonary endothelial cells (ECs) via the angiotensin‐converting enzyme 2 receptors with sustained infection resulting in the destruction of ECs and vascular leak, causing tissue edema and thromboinflammation.^1^ Patients with severe COVID‐19 often present with refractory hypoxemia arising from EC damage and pulmonary vasculature dysfunction. Severe cases have been associated with the upregulation of prothrombotic/inflammatory proteins. Two members of the transforming growth factor‐β family, bone morphogenetic protein 9 (BMP9) and BMP10, are recognized as vascular quiescence factors that guard against endothelial dysfunction. We have previously established that BMP9 protects against excess endothelial permeability associated with pulmonary arterial hypertension.^2^ Furthermore, BMP9 administration protected mice from lung injury and vascular permeability in a murine experimental model of acute lung injury (ALI). In addition, plasma BMP9 concentrations were shown to be markedly reduced in both patients with sepsis and endotoxemic mice.^3^ Given that COVID‐19 is a cause of acute respiratory distress syndrome (ARDS), we investigated circulating concentrations of BMP9 and prodomain‐BMP10 (pBMP10) in a cohort of patients with COVID‐19.

## METHODS

Following informed consent, plasma was obtained from hospital inpatients at Cambridge University Hospitals NHS Trust, Cambridge, UK with a confirmed diagnosis of COVID‐19 via a nucleic acid amplification test, between October 2, 2020 and February 19, 2021. Healthy controls (*n* = 29) were recruited through the University of Cambridge COVID‐19 asymptomatic screening program. Recruitment of inpatients at Cambridge University Hospital NHS Foundation Trust and controls was undertaken by the NIHR Cambridge Clinical Research Facility Outreach Team and the NIHR BioResource research nurse team. Ethical approval was provided by the East of England—Cambridge Central Research Ethics Committee (REC ref. no.: 17/EE/0025 for the NIHR BioResource Research Tissue Bank). Structured electronic health record data were extracted from the EpiCov Database specifically created to support COVID‐19 relevant monitoring and research. The EpiCov database is regulated under NHS Research Ethics Committee (REC) permission (ref. no.: 20/EE/0270). Patients with COVID‐19 were recruited at or soon after admission to the hospital and were divided into two categories of clinical severity based upon the WHO clinical progression scale^4^: 4, 5, 6 = hospitalized with moderate/severe disease and oxygen therapy via mask, nasal prongs or noninvasive ventilation (*n* = 49); and 7, 8, 9 = hospitalized with severe disease requiring assisted ventilation (*n* = 22) (Table 1). Healthy controls were designated 0.

**Table 1:** Clinical features of study participants.

	WHO clinical progression scale group		
	0	4, 5, 6	7, 8, 9
Participants	29	49	22
Age (years), median (SD)	29.0 (12.7)	60.0 (11.6)	65.5 (8.5)
Sex (% male)	100	100	100
Length of time in hospital (days), median (SD)	NA	10.3 (14.0)	33.4 (24.9)
Deceased in hospital (%)	NA	8.2	45.5
Days from hospital admission to sample receipt (days), median (SD)	NA	4.1 (11.8)	13.3 (7.7)
Alanine transaminase (U/L), median (SD)	NA	64.0 (106.1)	60.0 (161.7)
Albumin (g/L), median (SD)	NA	25.0 (4.6)	21.0 (4.4)
Bilirubin (µmol/L), median (SD)	NA	9.0 (6.2)	7.5 (100.0)
Creatinine (µmol/L), median (SD)	NA	69.0 (51.9)	75.0 (48.4)
Sodium (mmol/L), median (SD)	NA	136.0 (3.8)	142.5 (5.9)
Urea (mmol/L), median (SD)	NA	7.3 (4.5)	13.6 (7.6)
MELD score	NA	8.7 (3.7)	9.0 (5.6)
Comorbidities			
Cardiovascular disease	0	22	13
Hypertension	0	14	7
Endocrine disease	0	19	11
Diabetes	0	13	5
Respiratory disease	2	17	7
Renal disease	0	6	7
Immunosuppression	0	5	6
Hepatic disease	1	1	3
Hematological disease	2	9	2
Rheumatological disease	0	9	2
Central nervous system disease	0	8	1
Gastrointestinal disease	1	4	1
Oncological disease	0	8	0
COVID therapies			
Baricitinib	NA	6	0
Dapagliflozin	NA	3	1
Dexamethasone	NA	49	21
Hydrocortisone	NA	9	10
Remdesivir	NA	16	6
Tocilizumab	NA	1	4
Days on dexamethasone before sample receipt (days), median (SD)	NA	14.0 (7.1)	10.0 (6.4)

Abbreviations: COVID, coronavirus disease; MELD, Model for End‐Stage Liver Disease; NA, not applicable; WHO, World Health Organization.

Blood samples were drawn into EDTA blood tubes (BD Biosciences) upon study enrollment and plasma samples were processed on the same day as receipt. Acellular banked plasma aliquots were stored at −80°C. A stock aliquot per participant was then defrosted and subaliquoted for this study, meaning the plasma provided had one freeze–thaw cycle. Enzyme‐linked immunosorbent assays (ELISAs) for BMP9 and pBMP10 were conducted as previously described.^5^, ^6^ The soluble endoglin Quantikine assay (R&D Systems) was performed according to the manufacturer's instructions. A range of angiogenesis and vascular injury biomarkers were measured using three MesoScale Discovery multiplex immunoassay panels following the manufacturer's protocol: V‐PLEX Angiogenesis Panel 1 kit (fibroblast growth factor [FGF] (basic), placental growth factor [PlGF], Tie‐2, vascular endothelial growth factor‐A [VEGF‐A], VEGF‐C, VEGF‐D, and VEGFR‐1/Flt‐1); Human Vascular Injury I kit (E‐selectin, intercellular adhesion molecule‐3 [ICAM‐3], P‐selectin, and thrombomodulin) and V‐PLEX Vascular Injury Panel 2 Human kit (C‐reactive protein [CRP], ICAM‐1, serum amyloid A [SAA], and vascular cell adhesion molecule‐1 [VCAM‐1]).

## RESULTS

**Figure 1:**
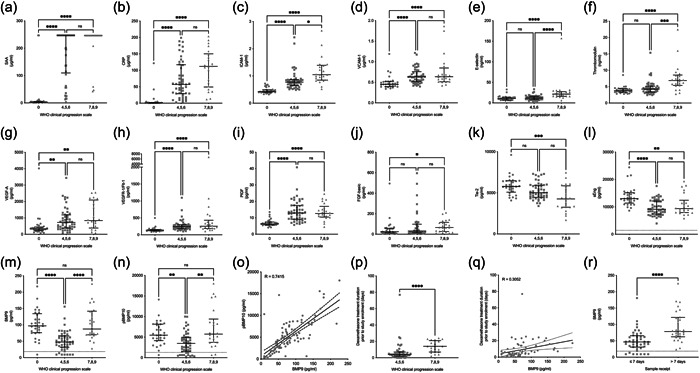
Circulating vascular injury and angiogenesis biomarkers after coronavirus disease 2019 (COVID‐19) infection. Plasma samples from healthy controls (0; *n* = 29), patients whose maximal respiratory support was supplemental oxygen (4, 5, 6; *n* = 49), and patients who required assisted ventilation (7, 8, 9; *n* = 22) were assessed for the following analytes using MesoScale Discovery multiplex immunoassays. (a) Serum amyloid A (SAA); (b) C‐reactive protein (CRP); (c) intercellular adhesion molecule 1 (ICAM‐1); (d) vascular cell adhesion molecule 1 (VCAM‐1); (e) E‐selectin; (f) thrombomodulin; (g) vascular endothelial growth factor A (VEGF‐A); (h) VEGFR1/Flt‐1; (i) placental growth factor (PlGF); (j) fibroblast growth factor (basic) (FGF(b)); (k) Tie‐2; (l) soluble endoglin (sENG) enzyme‐linked immunosorbent assay (ELISA); (m) bone morphogenetic protein 9 (BMP9) ELISA; (n) prodomain BMP10 (pBMP10) ELISA; (o) Pearson's correlation of circulating BMP9 and pBMP10 levels. *p* = 0.7415; (p) number of days patients were administered dexamethasone treatment before sample receipt; (q) Pearson's correlation of circulating BMP9 levels and dexamethasone treatment length. *p* = 0.3052; (r) circulating BMP9 levels of patient groups separated upon the time taken to be enrolled into the study. Sample receipt ≤7 days versus >7 days. All data presented as median–interquartile range. Kruskal–Wallis test. Dunn's multiple comparison test. ns, not significant; WHO, World Health Organization. *p* Values = *0.05; **0.01; ***0.001; ****0.0001.

We confirmed many of the vascular injury and proangiogenesis markers previously reported to be associated with disease severity and mortality in COVID‐19. First, SAA and CRP were significantly elevated in all patients with COVID‐19 (Figure 1a and b, respectively).

Consistent with previous COVID‐19 biomarker studies, plasma concentrations of the proinflammatory adhesion molecules ICAM‐1, VCAM‐1, and E‐selectin were significantly elevated, and associated with disease severity.^7^ Both ICAM‐1 and VCAM‐1 concentrations were elevated in nonventilated (4, 5, 6) and ventilated (7, 8, 9) patients (Figure 1c and d, respectively). Additionally, ICAM‐1 was significantly increased in ventilated patients compared to nonventilated (Figure 1c). Similarly, elevated concentrations of E‐selectin were only seen in severe (7, 8, 9) patients (Figure 1e). Elevated plasma thrombomodulin concentration correlated with decreased rates of hospital discharge and survival after COVID‐19 infection,^8^ and was significantly increased in severe (7, 8, 9) disease (Figure 1f).

We also assessed the circulating concentrations of proangiogenesis markers that have been reported as predictive of COVID‐19 disease and severity.^9^, ^10^ VEGF pathway activation is associated with ARDS. Both nonventilated (4, 5, 6) and ventilated patients (7, 8, 9) had elevated plasma concentrations of VEGF‐A (Figure 1g). Increased VEGFR‐1/Flt‐1 has previously been reported to correlate with disease severity, but in this cohort both nonventilated (4, 5, 6) and ventilated (7, 8, 9) patients had significantly increased concentrations of the VEGF receptor, VEGFR‐1/Flt‐1 (Figure 1h).^11^ PlGF release was increased in both nonventilated (4, 5, 6) and ventilated (7, 8, 9) patients (Figure 1i), and FGF (basic) was significantly increased in ventilated patients (Figure 1j). The plasma concentration of the vessel maturity receptor, Tie‐2 (Tek),^12^ was markedly decreased in ventilated patients compared to healthy controls and nonventilated patients (Figure 1k). Interestingly, pro‐BMP9 treatment in a murine model of ALI increased the transcriptional expression of Tek.^3^ Soluble endoglin (sEng) is associated with inflammation/endothelial dysfunction,^13^ and increased sEng levels have been reported in people who do not survive COVID‐19 infection.^14^ Surprisingly, sEng concentrations in this cohort were decreased in both nonventilated (4, 5, 6) and ventilated (7, 8, 9) subjects (Figure 1l). A reported increase of sEng was observed in nonsurvivors of COVID‐19.^14^ In fact, sEng concentrations in survivors increased 14 days after study inclusion but were actually lower in patients than healthy controls at Day 0.^14^ Our plasma samples were collected as close to hospital admission as possible, and possibly closer to the initial infection. Therefore, secretion of sEng may increase over the duration of COVID‐19, and may be associated with sustained endothelial dysfunction in patients with persistent illness.

The data from our cohort expands the previous evidence that biomarkers associated with vascular injury and EC dysfunction are increased in COVID‐19. Given the clear association between COVID‐19 severity and inflammation/endothelial dysfunction, we hypothesized that endothelial‐selective BMP ligands may be novel biomarkers for endothelial injury in COVID‐19. BMP9 plasma concentrations were significantly decreased in only nonventilated (4, 5, 6) patients compared to ventilated (7, 8, 9) patients and healthy controls (Figure 1m). Similarly, circulating pBMP10 concentrations were reduced in nonventilated patients (Figure 1n). As previously reported, there was a strong correlation between plasma BMP9 and pBMP10 concentrations (Figure 1o).^6^

This is the first report that circulating concentrations of BMP9 and pBMP10 are decreased during COVID‐19. This is perhaps unsurprising given the association of inflammation and vascular dysfunction with BMP9 and pBMP10. In fact, BMP9 and pBMP10 have been shown to inhibit chemokine (C–C motif) ligand 2 secretion by vascular ECs, while endogenous circulating BMP9 is elevated in inflammation.^3^, ^15^ Induced endotoxemia by lipopolysaccharide (LPS) treatment revealed that liver BMP9 expression was reduced by 3–6 h, returning to normal levels by 18–24 h.^3^ Plasma BMP9 levels gradually declined 24 h post‐LPS administration, but whether plasma BMP9 remained reduced was not investigated.^3^ Therefore, it is plausible that circulating levels of BMP9 and pBMP10 are reduced by systemic inflammation. However, in this study, BMP9 and pBMP10 did not correlate with CRP and SAA, markers of systemic inflammation. Although not addressed here, the release of neutrophil elastase (NE) could be assessed and correlated with BMP9 plasma levels to further investigate the role of inflammation. It is known that neutrophils isolated from COVID‐19 patients have increased NE release, and BMP9 is a substrate of NE.^3^, ^16^ However, it is still unclear whether the downregulation of BMP9 and pBMP10 is due to SARS‐CoV‐2 infection or associated inflammatory factors.

Interestingly, plasma concentrations did not correlate with disease severity as individuals who required mechanical ventilation had similar plasma concentrations to control subjects. The principal limitations of our study are the small number of patients recruited into the ventilated (7, 8, 9) cohort and the lack of longitudinal follow‐up. Upon examination of the COVID‐19 medications prescribed, we discovered that all ventilated patients were administered dexamethasone for significantly longer before study enrollment than those individuals requiring noninvasive oxygen therapy (Figure 1p). Successful treatment of COVID‐19 with dexamethasone was originally highlighted by the Recovery Trial in the United Kingdom,^17^ and we observed elevated BMP9 concentrations mildly correlated with the duration of dexamethasone treatment (Figure 1q).

Furthermore, due to the nature of patient recruitment, we also observed that several individuals in both the nonventilated and ventilated groups were not enrolled in the study until 7 days after hospital admission. Interestingly, plasma BMP9 (and pBMP10; data not shown) concentrations were decreased in patients recruited within 7 days of hospital admission, when compared to those recruited after 7 days (Figure 1r). We therefore cannot rule out that the administration of COVID‐19 therapies (Table 1) might lead to the normalization of BMP9 (or pBMP10) concentrations. We hypothesize that plasma BMP9 and pBMP10 concentrations may be novel biomarkers of endothelial injury observed in hospitalized patients with COVID‐19. However, longitudinal analysis would be required to determine whether these could predict disease severity and clinical outcome.

## AUTHOR CONTRIBUTIONS

Benjamin J. Dunmore designed, performed, and analyzed the experiments, and wrote the manuscript; Paul D. Upton performed the ELISAs and contributed to writing the manuscript; Kate Auckland analyzed clinical data; R. J. Samant analyzed clinical data; CITIID‐NIHR BioResource COVID‐19 Collaboration enrolled patients, collected/processed samples, and analyzed clinical data. The EpiCov database collated electronic health record data. Paul A. Lyons devised and supervised the collection and processing of COVID‐19 samples. Kenneth G. C. Smith devised and supervised the collection and processing of COVID‐19 samples. Stefan Gräf devised and supervised the collation of electronic health records and supervised the analysis of clinical data. Charlotte Summers supervised the analysis of clinical data and wrote the manuscript. Nicholas W. Morrell devised the study and wrote the manuscript.

## CITIID‐NIHR BioResource COVID‐19 Collaboration

Stephen Baker^2,6^, John Bradley^1,3,6,10,14,^ Patrick Chinnery^3,22,23^, Daniel Cooper^10, 24^, Gordon Dougan^2,6^, Ian Goodfellow^7^, Ravindra Gupta^2,6,12,15^, Nathalie Kingston^3,4^, Paul J. Lehner^2,6,12^, Paul A. Lyons^2,6^, Nicholas J. Matheson^2,6,12,26^, Caroline Saunders^9^, Kenneth G. C. Smith^2,6^, Charlotte Summers^6,11,25^, James Thaventhiran^18^, M. Estee Torok ^6,12,13^, Mark R. Toshner^6,8,25^, Michael P. Weekes^2,6,12,27^, Gisele Alvio^9^, Sharon Baker^9^, Areti Bermperi^9^, Karen Brookes^9^, Ashlea Bucke, Jo Calder, Laura Canna, Cherry Crucusio, Isabel Cruz^9^, Ranalie de Jesus^9^, Katie Dempsey^9^, Giovanni Di Stephano^9^, Jason Domingo^9^, Anne Elmer^9^, Julie Harris, Sarah Hewitt, Heather Jones^9^, Sherly Jose^9^, Jane Kennet, Yvonne King,, Jenny Kourampa^9^, Emily Li, Caroline McMahon^9^, Anne Meadows, Vivien Mendoza^9^, Criona O'Brien, Charmain Ocaya^9^, Ciro Pasquale^9^, Marlyn Perales^9^, Jane Price, Rebecca Rastall, Carla Ribeiro^9^, Jane Rowlands, Valentina Ruffolo, Hugo Tordesillas, Phoebe Vargas^9^, Bensi Vergese^9^, Laura Watson^9^, Jieniean Worsley^9^, Julie‐Ann Zerrudo^9^, Laura Bergamashi^2,6^, Ariana Betancourt, Georgie Bower, Ben Bullman, Chiara Cossetti, Aloka De Sa, Benjamin J. Dunmore^6,29^, Maddie Epping, Stuart Fawke, Stefan Gräf ^3,6,29^, Richard Grenfell, Andrew Hinch, Josh Hodgson, Christopher Huang, Oisin Huhn, Kelvin Hunter^2,6^, Isobel Jarvis, Emma Jones, Maša Josipović, Ekaterina Legchenko, Daniel Lewis, Joe Marsden, Jennifer Martin, Federica Mescia^2,6^, Ciara O'Donnell, Ommar Omarjee, Marianne Perera, Linda Pointon, Nicole Pond, Nathan Richoz, Nika Romashova, Natalia Savinykh, Rahul Sharma, Joy Shih, Mateusz Strezlecki, Rachel Sutcliffe, Tobias Tilly, Zhen Tong, Carmen Treacy, Lori Turner, Jennifer Wood, Marta Wylot, John Allison^3,4^, Heather Biggs^3,17^, John R. Bradley^1,3,6,10,14^, Helen Butcher^3,5^, Daniela Caputo^3,5^, Matt Chandler^3,5^, Patrick Chinnery^3,22,23^, Debbie Clapham‐Riley^3,5^, Eleanor Dewhurst^3,5^, Christian Fernandez^3,^ Anita Furlong^3,5^, Barbara Graves^3,5^, Jennifer Gray^3,5^, Sabine Hein^3,5^, Tasmin Ivers^3,5^, Emma Le Gresley^3,5^, Rachel Linger^3,5^, Mary Kasanicki^3,10^, Rebecca King^3,5^, Nathalie Kingston^3,4^, Sarah Meloy^3,5^, Alexei Moulton^3,5^, Francesca Muldoon^3,5^, Nigel Ovington^3,4^, Sofia Papadia^3,5^, Christopher J. Penkett^3,4^, Isabel Phelan^3,5^, Venkatesh Ranganath^3,4^, Roxana Paraschiv^3,4^, Abigail Sage^3,5^, Jennifer Sambrook^3,4^, Ingrid Scholtes^3,5^, Katherine Schon^3,16,17^, Hannah Stark^3,5^, Kathleen E. Stirrups^3,4^, Paul Townsend^3,4^, Neil Walker^3,4^, Jennifer Webster^3,5^, Mayurun Selvan^28^, Petra, Polgarova^11^, Sarah L. Caddy^2,6^, Laura G. Caller^19,20^, Yasmin Chaudhry^7^, Martin D. Curran^21^, Theresa Feltwell^6^, Stewart Fuller^19^, Iliana Georgana^7^, Grant Hall^7^, William L. Hamilton^6,12,13^, Myra Hosmillo^7^, Charlotte J. Houldcroft^6^, Rhys Izuagbe^7^, Aminu S. Jahun^7^, Fahad A. Khokhar^2,6^, Anna G. Kovalenko^7^, Luke W. Meredith^7^, Surendra Parmar^21^, Malte L. Pinckert^7^, Anna Yakovleva^7^, Emily C. Horner^18^, Lucy Booth^18^, Alexander Ferreira^18^, Rebecca Boston^18^, Robert Hughes^18^, Juan Carlos Yam Puc^18^, Nonantzin Beristain‐Covarrubias^18^, Maria Rust^18^, Thevinya Gurugama^18^, Lihinya Gurugama^18^, Thomas Mulroney^18^, Sarah Spencer^18^, Zhaleh Hosseini^18^, Kate Williamson^18^, Neda Farahi^6,29^

^1^NIHR Cambridge Biomedical Research Centre, Cambridge Biomedical Campus, Cambridge, UK

^2^Cambridge Institute of Therapeutic Immunology and Infectious Disease (CITIID), Jeffrey Cheah Biomedical Centre, Cambridge Biomedical Campus, Cambridge, UK

^3^NIHR BioResource, Cambridge University Hospitals NHS Foundation Trust, Cambridge Biomedical Campus, Cambridge, UK

^4^Department of Haematology, School of Clinical Medicine, University of Cambridge, Cambridge Biomedical Campus, Cambridge, UK

^5^Department of Public Health and Primary Care, School of Clinical Medicine, University of Cambridge, Cambridge Biomedical Campus, Cambridge, UK

^6^Department of Medicine, School of Clinical Medicine, University of Cambridge, Cambridge Biomedical Campus, Cambridge, UK

^7^Division of Virology, Department of Pathology, University of Cambridge, Cambridge, UK

^8^Royal Papworth Hospital NHS Foundation Trust, Cambridge, UK

^9^Cambridge Clinical Research Centre, Addenbrooke's Hospital, Cambridge University Hospitals NHS Foundation Trust, Cambridge, UK

^10^Addenbrooke's Hospital, Cambridge University Hospitals NHS Foundation Trust, Cambridge Biomedical Campus, Cambridge, UK

^11^Intensive Care Unit, Addenbrooke's Hospital, Cambridge University Hospitals NHS Foundation Trust, Cambridge Biomedical Campus, Cambridge, UK

^12^Department of Infectious Diseases, Addenbrooke's Hospital, Cambridge University NHS Hospitals Foundation Trust, Cambridge, UK

^13^Department of Microbiology, Addenbrooke's Hospital, Cambridge University NHS Hospitals Foundation Trust, Cambridge, UK

^14^Department of Renal Medicine, Addenbrooke's Hospital, Cambridge University Hospitals NHS Foundation Trust, Cambridge, UK

^15^Africa Health Research Institute, Durban, South Africa

^16^Clinical Genetics, Addenbrooke's Hospital, Cambridge University Hospitals NHS Foundation Trust, Cambridge, UK

^17^Department of Clinical Neurosciences, School of Clinical Medicine, University of Cambridge, Cambridge Biomedical Campus, Cambridge, UK

^18^MRC Toxicology Unit, Gleeson Building, Tennis Court Road, Cambridge, UK

^19^University of Cambridge, Cambridge, UK

^20^The Francis Crick Institute, London, UK

^21^Public Health England, Clinical Microbiology and Public Health Laboratory, Cambridge, UK

^22^Department of Clinical Neurosciences, School of Clinical Medicine, University of Cambridge, Cambridge Biomedical Campus, Cambridge, UK

^23^Medical Research Council Mitochondrial Biology Unit, Cambridge Biomedical Campus, Cambridge, UK

^24^Global and Tropical Health Division, Menzies School of Health Research and Charles Darwin University, Darwin, Northern Territory, Australia

^25^Heart and Lung Research Institute, Cambridge Biomedical Campus, Cambridge, UK

^26^NHS Blood and Transplant, Cambridge, UK

^27^Cambridge Institute for Medical Research, Biomedical Campus, Hills Rd, Cambridge UK

^28^Department of Respiratory Medicine, Cambridge University Hospitals NHS Foundation Trust, Cambridge, UK

^29^Heart and Lung Research Institute, University of Cambridge, Cambridge, CB2 0BB, UK

## EpiCov Database Collaboration

Vince Taylor^1^, Helen Street^1^, Adam Loveday^1^, Habeebat Ibraheem^1^, Jacob Letowski^1^, Peter Driscoll^1^, Afzal Chaudhry^1^, Mark Sharpley^2^, Guilherme Balzana^2^, Wojciech J. Turek^2^, Stuart J. Rankin^2^, Paul Calleja^2^, Nicholas S. Gleadall^1,3,4^, Connor Rochford^1,3,4^, John R. Bradley^1,3,4^, Willem H. Ouwehand^1,3,4^, Stefan Gräf^1,3,4^

^1^Cambridge University Hospitals NHS Foundation Trust, Cambridge, UK

^2^Research Computing Service, University of Cambridge, Cambridge, UK

^3^NIHR Cambridge Biomedical Research Centre, Cambridge Biomedical Campus, Cambridge, UK

^4^University of Cambridge, Cambridge, UK

## CONFLICTS OF INTEREST STATEMENT

Paul D. Upton is a founder of, and scientific advisor to, Morphogen‐IX Ltd. Nicholas W. Morrell is a founder and CEO of Morphogen‐IX Ltd. Paul D. Upton and Nicholas W. Morrell have published US (US10336800) and EU (EP3166628B1) patents entitled: “Therapeutic Use of Bone Morphogenetic Proteins.” The remaining authors declare no conflicts of interest.

## ETHICS STATEMENT

The ethics for this study were approved by the Cambridge Central Research Ethics Committee and the NHS Research Ethics Committee. All patients provided informed written consent.

## Data Availability

The data that support the findings of this study are available from the corresponding author upon reasonable request.
